# Stem Cell Therapies in Canine Cardiology: Comparative Efficacy, Emerging Trends, and Clinical Integration

**DOI:** 10.3390/biom15030371

**Published:** 2025-03-04

**Authors:** Ahmed Farag, Hanan Hendawy, Mahmoud H. Emam, Mizuki Hasegawa, Ahmed S. Mandour, Ryou Tanaka

**Affiliations:** 1 Faculty of Agriculture, Veterinary Teaching Hospital, Tokyo University of Agriculture and Technology, Tokyo 183-8509, Japan; 2 Department of Surgery, Anesthesiology, and Radiology, Faculty of Veterinary Medicine, Zagazig University, Zagazig 44519, Egypt; 3 Department of Veterinary Surgery, Faculty of Veterinary Medicine, Suez Canal University, Ismailia 41522, Egypt; 4 Animal Medicine Department, Faculty of Veterinary Medicine, Zagazig University, Zagazig 44519, Egypt; 5 Department of Animal Medicine (Internal Medicine), Faculty of Veterinary Medicine, Suez Canal University, Ismailia 41522, Egypt

**Keywords:** canine cardiology, stem cell therapy, mesenchymal stem cells, cardiac regeneration, veterinary cardiology

## Abstract

Cardiovascular diseases are a leading cause of morbidity and mortality in dogs, with limited options available for reversing myocardial damage. Stem cell therapies have shown significant potential for cardiac repair, owing to their immunomodulatory, antifibrotic, and regenerative properties. This review evaluates the therapeutic applications of mesenchymal stem cells (MSCs) derived from bone marrow, adipose tissue, and Wharton’s jelly with a focus on their role in canine cardiology and their immunoregulatory properties. Preclinical studies have highlighted their efficacy in enhancing cardiac function, reducing fibrosis, and promoting angiogenesis. Various delivery methods, including intracoronary and intramyocardial injections, are assessed for their safety and efficacy. Challenges such as low cell retention, differentiation efficiency, and variability in therapeutic responses are also discussed. Emerging strategies, including genetic modifications and combination therapies, aim to enhance the efficacy of MSCs. Additionally, advances in delivery systems and regulatory frameworks are reviewed to support clinical translation. This comprehensive evaluation underscores the potential of stem cell therapies to revolutionize canine cardiovascular disease management while identifying critical areas for future research and clinical integration.

## 1. Introduction

### 1.1. Overview of Cardiovascular Diseases

Cardiovascular diseases are a significant health challenge in dogs, contributing to roughly 8% of mortalities in companion animals [1,2,3]. Estimates suggest that 10–15% of dogs seen in veterinary clinics are diagnosed with some form of heart disease, most of which are acquired rather than congenital [4]. Canine cardiac diseases can be broadly classified into valvular, myocardial, and pericardial types [1]. The most prevalent acquired cardiac condition is myxomatous mitral valve disease (MMVD), affecting approximately 75% of cases and leading to degenerative changes in the mitral valve [5]. Dilated cardiomyopathy (DCM) is a common cardiac disease in medium- to large-sized dog breeds, characterized by cardiac chamber dilation, systolic dysfunction, and, in some cases, ventricular arrhythmias [6]. It is a leading cause of congestive heart failure (CHF) and sudden cardiac death in these dogs, particularly in breeds such as Doberman Pinschers, Boxers, and Irish Wolfhounds [7]. In most cases, DCM has a genetic basis but is often detected later in life due to its insidious onset. This condition carries a grave prognosis, with affected dogs succumbing to progressive heart failure refractory to treatment or sudden death, likely due to malignant arrhythmia [8]. Additionally, systemic factors, such as hypothyroidism and nutritional deficits, can predispose dogs to DCM-like symptoms [1,9]. Although rare, hypertrophic cardiomyopathy (HCM) in dogs presents with left ventricular hypertrophy (LVH) due to systemic conditions, including hyperadrenocorticism and systemic hypertension [10,11]. Pericardial disorders, encompassing pericardial effusion and cardiac tamponade, account for around 8% of canine cardiac diseases, while cardiac tumors such as hemangiosarcoma and lymphoma remain relatively uncommon, seen in 0.12–4.33% of cases [12,13].

Dogs are increasingly recognized as valuable models for studying human diseases due to their physiological and genetic similarities with humans. This relevance is especially pronounced in cardiovascular research, where dogs naturally develop conditions such as heart failure, mitral valve disease, and arrhythmias, mirroring those seen in humans [14]. The molecular composition of canine ventricular tissue closely resembles that of the human heart, making dogs one of the most predictive preclinical species for assessing human electrocardiographic responses. Their cardiac conduction system shares similarities with humans in ion channel distribution and function, leading to comparable ECG morphology and conduction times [15]. These characteristics enable canine models, such as ventricular wedge preparations, to effectively predict proarrhythmic drug effects [16]. Additionally, the duration of key ECG elements in dogs, such as the PR interval (60–130 ms), QRS complex (30–50 ms), and QT interval (150–250 ms), falls within a comparable range to human values when adjusted for heart rate differences [17,18,19]. Furthermore, inherited arrhythmias in dogs provide valuable models for studying primary arrhythmic conditions, reinforcing the translational significance of canine electrophysiological research [20]. This natural occurrence allows researchers to explore disease progression and potential treatments in a clinically relevant setting, offering insights that laboratory-induced models may not capture.

### 1.2. Stem Cell Therapy: A Promising Approach

Mesenchymal stem cell therapy has gained attention for the treatment of heart disease due to the cells’ immunomodulatory, antifibrotic, and regenerative properties [21,22,23]. Numerous clinical trials are currently underway to investigate its potential for treating heart failure in humans [24]. MSCs can be derived from various sources, including umbilical cord, amniotic fluid, placenta, adipose tissue, joint synovium, synovial fluid, dental pulp, endosteum, and periosteum [25,26]. Among these, bone marrow and adipose tissue are the most commonly utilized sources for mesenchymal stem cells in musculoskeletal applications [27]. However, these methods of sampling are invasive and pose challenges, particularly in veterinary medicine [28].

In in vitro investigations, mesenchymal stem cells (MSCs) can differentiate into mesodermal lineage cells, including adipocytes, chondrocytes, and osteocytes [29,30]. Treatment with MSCs in myocardial infarction and heart failure models has recorded noticeable improvements in fibrosis, scar size, cardiomyocyte apoptosis, and cardiac function, including enhancements in ejection fraction and end-systolic volume [31]. Additionally, MSC therapy has shown a wide safety margin, with no observed cases of acute infusion toxicity [32]. MSC therapy has shown significant promise in treating cardiovascular diseases in small animals by improving cardiac function. The mechanisms behind these benefits are primarily attributed to the activation of endogenous repair processes, including immune regulation, enhanced tissue perfusion, inhibition of fibrosis, and the stimulation of resident cardiac cells. Although rare, studies in animal models have reported instances where MSCs transdifferentiate into cardiomyocytes and vascular components, further contributing to heart repair [33]. However, despite these advancements, complete cardiac recovery remains an elusive goal in both preclinical and clinical settings. To achieve more comprehensive cardiac regeneration, innovative approaches to MSC therapy are being explored. Current research focuses on combining MSCs with other cell types, incorporating them into biomaterials, or preconditioning and genetically modifying the cells to enhance the secretion of therapeutic paracrine factors, such as exosomes, growth factors, and microRNAs [34,35,36]. These strategies aim to increase the therapeutic efficacy of MSCs, addressing the limitations observed in earlier studies and bringing stem cell therapy closer to being a viable long-term solution for cardiac repair.

MSCs exhibit diverse therapeutic effects, largely influenced by their production, handling, and administration methods. Their clinical efficacy depends not only on their inherent regenerative and immunomodulatory properties but also on factors such as metabolic activity, disease stage, and patient-specific receptivity [37,38]. However, current MSC production methods often adopt a generalized approach, using the same process for diverse conditions like graft-versus-host disease, myocardial infarction, and lung injury, despite each condition requiring distinct therapeutic mechanisms. Optimizing MSC production by tailoring them for specific medical indications has the potential to enhance treatment efficacy. This process involves selecting MSCs based on their functional responses to relevant disease environments, such as testing their secretory profiles in vitro to identify donors with optimal bioactive molecule expression [39]. For instance, a clinical trial investigating MSC therapy for cystic fibrosis selected donor MSCs based on their ability to secrete antibiotic proteins and inflammatory mediators in response to bacterial exposure [37]. Additionally, preconditioning MSCs in culture using inflammatory mediators, such as IL-1 for rheumatoid arthritis, can enhance their adaptive response to in vivo conditions, ultimately improving therapeutic outcomes [40]. These strategies represent a shift toward personalized medicine, aiming to optimize MSC responsiveness and improve clinical efficacy, while also potentially expediting regulatory approval processes [37].

### 1.3. Scope and Objectives of the Review

While various MSC sources, such as placental and dental pulp-derived MSCs, have demonstrated potential in regenerative medicine, their application in canine cardiology remains limited. Placental MSCs possess strong immunomodulatory and angiogenic properties; however, their use in veterinary cardiology is restricted due to the lack of sufficient preclinical and clinical studies specifically focused on dogs [41]. Likewise, dental pulp-derived MSCs exhibit promising neurogenic and osteogenic potential, but their role in cardiac regeneration has not been extensively explored, with limited data supporting their efficacy in myocardial repair [42]. Furthermore, challenges related to large-scale isolation, donor variability, and standardization present additional barriers to their integration into veterinary cardiac therapies [43]. Given these constraints, this review primarily focuses on MSC sources with well-documented research in canine cardiology, including bone marrow, adipose-derived, and Wharton’s jelly-derived MSCs, which have demonstrated significant potential in cardiac repair and functional recovery [33].

This review provides a comprehensive assessment of the current and emerging applications of stem cell therapies in canine cardiology. By focusing on the therapeutic potential of MSCs from various sources, it evaluates their impact on myocardial repair and functional recovery in dogs with conditions such as myocardial infarction and chronic heart failure. Additionally, this review compares different stem cell delivery methods including intracoronary, intramyocardial, and retrograde coronary venous infusion to optimize therapeutic efficacy. By addressing the challenges and limitations associated with clinical applications, this review aims to enhance the integration of stem cell therapies into veterinary cardiology while identifying critical areas for future research and clinical advancements.

## 2. Efficacy of Stem Cell Therapies in Cardiology

### 2.1. Mesenchymal Stem Cells in Cardiac Regeneration

#### 2.1.1. Bone Marrow-Derived MSCs

Bone marrow-derived mesenchymal stem cells (BM-MSCs) have garnered significant attention in the field of cardiac regeneration due to their ability to differentiate into cardiomyocytes and support tissue repair. Their unique properties, including immunomodulation, paracrine signaling, and potential to promote angiogenesis, make them ideal candidates for treating various cardiac conditions, such as myocardial infarction (MI) and chronic heart failure. Research has demonstrated that BM-MSCs can improve cardiac function and enhance the regenerative processes in damaged myocardium, offering hope for more effective therapies in veterinary cardiology [44,45].

Many studies reported that delivering BM-MSCs to the hearts of post-MI patients significantly improves cardiac function, demonstrating therapeutic potential [46,47,48,49,50,51,52,53]. Transplantation of BM-MSCs has shown promising results in supporting cardiac regeneration, not only in myocardial infarction animal models but also in MI patients treated with intravenous BM-MSC therapy [54,55,56]. Nevertheless, the long-term effects remain uncertain. Despite numerous studies showing that stem cell transplantation can lead to neovascularization in infarct models, and while BM-MSCs in infarcted areas may differentiate into endothelial cells, smooth muscle cells, and cardiomyocytes, they can also lead to arrhythmias [57,58,59,60]. It is still unclear whether sustained clinical benefits in post-MI patients result directly from myocyte repair. Transplanting BM-MSCs into humans carries inherent risks due to their pluripotent nature. A key concern is the potential for unwanted differentiation, where BM-MSCs may transform into unintended cell types, leading to complications. For example, a study demonstrated that intramyocardial injection of total bone marrow cells into a rat model of acute myocardial infarction resulted in significant intramyocardial calcification in 28.5% of treated rats, highlighting the risks associated with unselected BM cell transplantation [61]. Additionally, MSC transplantation has been linked to tumorigenic potential, with some studies reporting the induction of sarcoma or facilitation of tumor growth [62]. Systemic administration of MSCs also poses challenges due to their immunosuppressive properties, which can increase susceptibility to severe infections. For instance, MSC therapy has been associated with an elevated risk of pneumonia-related mortality following allogeneic hematopoietic stem cell transplantation [63]. Furthermore, MSC therapy has been shown to alter lymphocyte populations, including reductions in CD4+ T-helper cells, CD8+ T-cells, and CD19+/CD20+ B-lymphocytes. These changes can impact immune system function, as evidenced by a decreased CD4/CD8 ratio and diminished B-lymphocyte populations in patients receiving MSC therapy [64,65]. These findings underscore the need for meticulous evaluation of BM-MSC therapies to mitigate adverse effects and ensure their safe application in clinical practice.

#### 2.1.2. Adipose-Derived Stem Cells

Adipose-derived stem cells (ASCs) have recently attracted considerable interest, as subcutaneous adipose tissue is plentiful and can be conveniently collected through liposuction—a less invasive and more comfortable procedure for donors compared to bone marrow aspiration. Adipose tissue contains a much higher concentration of stem cells than bone marrow (approximately 5% versus 0.01%), making it a highly accessible stem cell source [66]. Additionally, ASCs are reported to lack expression of class II major histocompatibility complexes, indicating that they may be suitable for both autologous and allogeneic transplantation [67,68,69].

Preclinical studies have demonstrated that both the stromal vascular fraction (SVF) and ASCs can enhance cardiac function, as evidenced by improvements in left ventricular ejection fraction (LVEF), fractional shortening, wall thickness, and contractility. Concurrently, these treatments have been associated with reductions in left ventricular end-diastolic diameter, left ventricular end-systolic diameter, and overall cardiac remodeling [70,71,72,73,74]. Motivated by these positive findings, several clinical trials have been initiated. One of the pioneering studies, the APOLLO trial, investigated the safety and feasibility of intracoronary infusion of ASCs in patients experiencing ST-elevation myocardial infarction. Outcomes reported at six months indicated successful revascularization, improved cardiac function, and a decrease in myocardial scarring. The ATHENA trial evaluated the safety and feasibility of intramyocardial ADRC therapy in chronic ischemic cardiomyopathy. Despite early termination due to non-ADRC-related adverse events, the results suggested potential benefits, including improved exercise capacity, better quality of life, and a trend toward fewer heart failure hospitalizations, though no significant changes in left ventricular function were observed [75,76].

#### 2.1.3. Wharton’s Jelly-Derived MSCs

Wharton’s jelly from the umbilical cord is emerging as an optimal source of allogeneic MSCs for cell therapy, given that it can be collected in large quantities from numerous births worldwide without pain or donor site complications. Wharton’s jelly-derived MSCs (WJ-MSCs) are not only easily isolated and cultured but also demonstrate a high proliferative rate and the ability to retain stemness across multiple passages in vitro. They are hypoimmunogenic, non-tumorigenic, and multipotent, while also being less heterogeneous than adult MSCs, making them highly suitable for therapeutic applications [77,78].

WJ-MSCs exhibit strong homing capabilities, allowing them to migrate efficiently to damaged tissues when administered systemically [79,80]. These MSCs are easier to harvest and expand compared to bone marrow-derived MSCs, and they demonstrate superior angiogenic and paracrine effects, which play a critical role in promoting tissue regeneration [81]. Additionally, since WJ-MSCs originate from neonatal donors, they display fewer features of cellular aging, further enhancing their therapeutic potential [81].

### 2.2. Cardiac Stem Cells (CSCs) and Their Potential

Cardiac stem cells (CSCs) present a promising source for cardiac regeneration therapy, as they are inherently programmed to produce cardiac tissue and enhance its viability, unlike other adult stem cells such as MSCs [82]. Theoretically, CSCs represent the most logical and potentially effective cell population for cardiac repair [83]. Cardiac regeneration through CSCs could be achieved by stimulating the natural turnover of myocardial cells [84].

Researchers have increasingly highlighted the regenerative potential of cardiac stem cells (CSCs) in repairing damaged heart tissue [85]. Initially discovered by Beltrami et al. [86], CSCs, particularly those expressing c-kit, have been shown to possess self-renewing, multipotent, and clonogenic properties. These cells can differentiate into various cardiac cell types, including cardiomyocytes, endothelial cells, and smooth muscle cells. Myocyte progenitor cells within the c-kit+ population exhibit a greater ability to generate cardiomyocytes, while vascular progenitors typically differentiate into endothelial and smooth muscle cells [87]. When injected into a damaged myocardium, CSCs have demonstrated the ability to restore cardiac structure and function [88]. Additionally, CSCs secrete cytokines and growth factors, such as VEGF, which promote angiogenesis and support cardiac repair through paracrine signaling [44,89].

Studies in dogs have shown that CSCs are capable of self-renewal and can differentiate into cardiomyocytes and vascular cells, contributing to the regeneration of damaged myocardium. Notably, canine CSCs express key factors like hepatocyte growth factor (HGF) and insulin-like growth factor 1 (IGF-1), which stimulate migration, proliferation, and survival of these cells in response to cardiac injury. Following myocardial infarction, the intramyocardial injection of CSCs has led to the formation of new myocytes and blood vessels within the infarcted region, promoting improved heart function and contractile performance [90]. An additional study explored whether large mammalian hearts contain cardiac progenitor cells capable of maintaining tissue health and regenerating damaged myocardium after infarction. Results revealed that the dog heart hosts self-renewing, clonogenic, and multipotent cardiac stem cells (CSCs) with hepatocyte growth factor (HGF)-c-Met and insulin-like growth factor 1 (IGF-1)-IGF-1 receptor systems, which, when activated, promote migration, proliferation, and survival of these cells. Following myocardial infarction in dogs, direct injections of HGF and IGF-1 into the myocardium activated resident CSCs, initiating the formation of new myocytes and coronary vessels within the damaged region. The newly developed cells expressed specific cardiac proteins, confirming their identity as functional cardiomyocytes, and contributed to a significant recovery of contractile performance in the heart. These findings suggest that therapies targeting CSC activation may offer a promising approach for myocardial repair after ischemic injury [91].

### 2.3. Effectiveness of Stem Cell Therapies in Canine Cardiac Models

**Figure 1:**
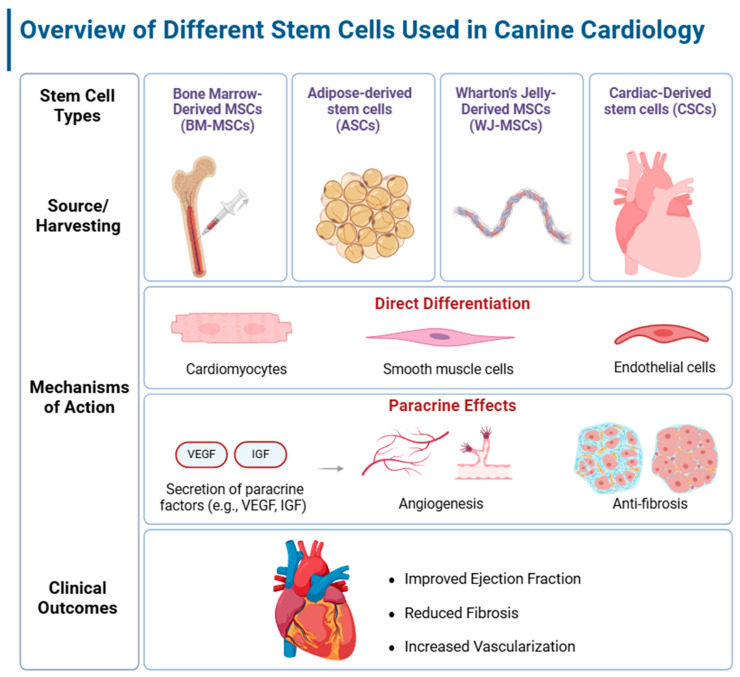
Overview of different stem cell sources used in canine cardiology, highlighting general therapeutic effects and mechanisms of action.

In canine cardiac models, MSCs derived from sources such as bone marrow, adipose tissue, and Wharton’s jelly have shown considerable potential in addressing heart diseases, including dilated cardiomyopathy (DCM) and chronic myocardial infarction (MI). Studies in dogs report promising improvements in cardiac function and fibrosis reduction. For instance, treating dogs with cardiomyogenic growth factor-pretreated autologous bone marrow MSCs post-MI significantly enhanced left ventricular function, evidenced by increased wall thickening and reduced wall motion abnormalities, suggesting effective cardiac repair [92] (Figure 1). Various stem cell delivery methods, such as intracoronary and intramyocardial injections, have also proven safe and effective in treating both acute and chronic MI in canine models. However, therapeutic outcomes may vary depending on the MSC source and the specific cardiac condition treated [93].

Limited studies comparing different stem cell types for myocardial repair have highlighted the superior therapeutic potential of bone marrow mononuclear cells (BMNCs) over MSCs. In a study by Mathieu et al. (2009), BMNC therapy in a canine model of chronic myocardial infarction resulted in significant improvements in cardiac function, including sustained enhancements in wall motion, increased cardiac contractility, and reduced infarct size. These outcomes were accompanied by favorable angiogenic changes, suggesting a robust regenerative environment. In contrast, MSC therapy showed only modest improvements, with no significant changes in cardiac histology [94]. Similarly, van der Bogt et al. (2008) demonstrated in a murine myocardial infarction model that BMNCs conferred superior survival and functional preservation compared to MSCs, with BMNC-treated animals exhibiting reduced left ventricular dilatation and better fractional shortening [95]. These studies underscore the greater efficacy of BMNCs in promoting cardiac recovery, suggesting that their mixed cellular composition may contribute to more effective myocardial repair than MSC-based therapies. Table 1 presents an overview of different stem cell types used, including their accessibility, clinical efficacy, safety, and mechanisms of action.

## 3. Delivery Methods for Stem Cell Therapies

When considering stem cell therapy for cardiac conditions, it is crucial to evaluate the delivery method to the myocardium. Since dilated cardiomyopathy (DCM) results in widespread myocardial dysfunction, a global distribution of a large number of stem cells is likely required to achieve clinical efficacy. Direct myocardial injection via thoracotomy is possible but highly invasive, requiring numerous injections to cover the entire left ventricular myocardium and excluding the interventricular septum [90,102]. Direct intramyocardial injection offers the advantage of precisely targeting cell delivery to the ischemic area, leading to higher cell retention compared to intracoronary injection. However, this method may also provoke inflammation, potentially compromising the survival of the engrafted cells [103,104]. Catheter-based intramyocardial injection into the endocardial surface is another option, but it may be limited to the more apical portions of the left ventricular myocardium, and it necessitates left heart catheterization [105,106]. Non-selective peripheral venous injection of stem cells is not a viable method due to the lack of targeted myocardial delivery [107,108].

Direct coronary arterial infusion allows for global distribution, but it carries the risk of microcirculatory obstruction, which could lead to embolic myocardial damage [90,109,110]. In a study by Vulliet et al. (2004), the safety of intra-coronary injection of mesenchymal stromal cells (MSCs) was assessed in healthy dogs. After injecting autologous MSCs into the left circumflex coronary artery, acute myocardial ischemia was observed, characterized by ST segment elevation and T wave changes. Seven days later, myocardial infarction and areas of fibroplasia and macrophage infiltration were noted. Elevated cardiac troponin I levels and collagen deposition were also detected [111].

Retrograde coronary venous delivery, however, offers a promising alternative for achieving widespread myocardial distribution. This approach involves accessing the cardiac veins via the coronary sinus using percutaneous cardiac catheterization and carries a lower risk of myocardial infarction compared to coronary arterial delivery [112,113]. Studies in experimental dogs [107,114] and porcine models of acute myocardial infarction [115] have demonstrated the safety and feasibility of retrograde coronary venous delivery, with cells successfully retained in the myocardium.

**Figure 2:**
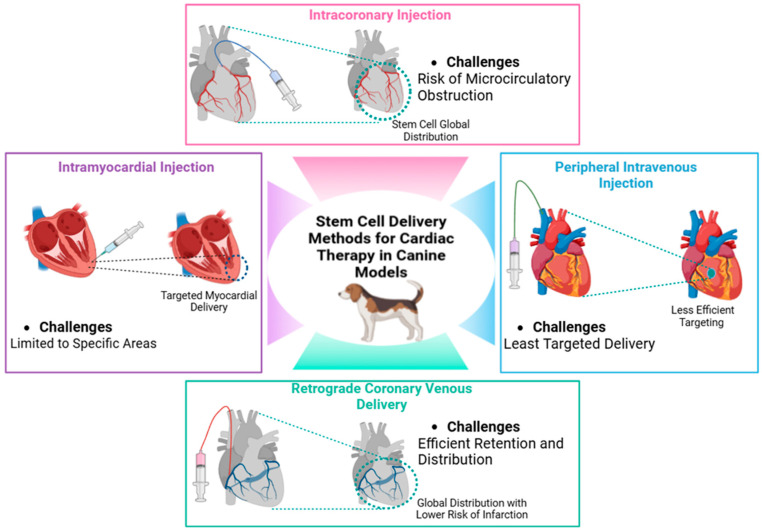
Overview of delivery methods for stem cell therapy in canine cardiology, including intramyocardial, intravenous, intracoronary, and retrograde coronary venous routes.

Intracoronary injection carries a significant risk of coronary embolism and typically results in low stem cell retention. To address these limitations, alternative delivery methods have been explored. For instance, ultrasound-mediated microbubble destruction using a micropump during intracoronary injection increases vascular permeability, thereby improving BM-MSC homing to cardiomyocytes and enhancing cardiac function [104]. Another approach involves percutaneous retrograde coronary injection combined with basic fibroblast growth factor (bFGF) [103], which has been shown to boost BM-MSC efficacy by enhancing MSC migration, viability, and differentiation into cardiomyocyte-like cells [116] (Figure 2).

## 4. Integrating Stem Cell Research with Clinical Practice

### 4.1. Bridging the Gap

The translation of stem cell research into clinical practice in small animal cardiology faces several significant barriers. Regulatory hurdles are one of the primary challenges, as the approval process for new therapies involves rigorous scrutiny to ensure safety and efficacy. This often results in lengthy and expensive trials, which can delay the availability of new treatments to veterinary practitioners [43]. Additionally, financial constraints can limit the ability of researchers and clinics to adopt new therapies, particularly when funding is sparse or unevenly distributed across institutions [117]. Technical limitations also pose considerable obstacles. The complexity of stem cell therapies, including the need for specialized equipment and expertise for cell isolation, culture, and transplantation, can be prohibitive for many veterinary clinics. Furthermore, ethical considerations, such as the source of stem cells and the welfare of animal subjects, add another layer of complexity to the adoption of these therapies. Balancing the potential benefits of stem cell treatments with ethical responsibilities toward animal welfare remains a critical concern [118,119]. Overcoming these barriers requires a concerted effort from researchers, clinicians, regulatory bodies, and funding agencies. Enhanced collaboration and communication between these stakeholders can help streamline the translation process, ensuring that promising research can more swiftly and safely transition into clinical applications. Developing robust frameworks for regulatory approval, securing adequate funding, and addressing technical and ethical challenges are essential steps towards integrating stem cell therapies into veterinary practice.

### 4.2. Clinical Guidelines and Protocols

The development of evidence-based clinical guidelines for stem cell therapies in small animal cardiology is essential for standardizing treatment and improving outcomes. Key recommendations include establishing interdisciplinary collaborations among experts in cardiology, stem cell biology, veterinary medicine, and regulatory affairs to ensure comprehensive and up-to-date guidelines. Standardized protocols for stem cell isolation, characterization, and administration should be based on rigorous preclinical and clinical studies, detailing patient selection, dosing regimens, and monitoring strategies. Emphasizing robust clinical trials and long-term studies will provide the necessary evidence to support these protocols. Additionally, knowledge dissemination through educational programs, training workshops, and accessible databases for tracking treatment outcomes is vital for successful implementation and continuous improvement of clinical practices. This collaborative and continuous learning approach will ensure the effective and safe use of stem cell therapies in small animal cardiology [120,121,122].

### 4.3. Case Studies Highlighting Stem Cell Therapies in Canine Cardiology

Stem cell therapies in canine cardiology have produced a range of outcomes, from promising improvements in cardiac function to cases requiring further investigation. One notable study evaluated the use of mouse HCN4-modified canine mesenchymal stem cells (cMSCs) in dogs with complete atrioventricular (AV) block. These modified cMSCs significantly improved cardiac function by increasing heart rates and impulse generation compared to the control group, and they also enhanced heart rate variability during exercise. The cMSCs were confirmed to survive and express the HCN4 protein in the heart, suggesting that gene-modified stem cells hold promise as biological pacemakers for treating AV block [123]. Similarly, the combination of endothelial progenitor cells (EPCs) and MSCs delivered via intracoronary infusion in dogs was shown to be both safe and feasible, with no significant adverse effects reported. Vital signs, electrocardiograms, and echocardiography remained stable, further supporting the potential clinical use of these cell combinations for treating coronary ischemia [124].

In another study, the administration of puppy deciduous teeth stem cells (pDSCs) in dogs with chronic valvular heart disease yielded promising results. Twenty client-owned dogs were treated either with standard heart failure therapy alone or combined with intravenous pDSC injections. Over a 60-day period, the pDSC-treated group showed significant improvements in left ventricular ejection fraction, ACVIM functional class, and overall quality of life, suggesting that pDSCs could be a valuable supplement to conventional treatments for valvular heart disease [101]. However, stem cell therapies are not without complications, as demonstrated in a study on Dobermanns diagnosed with dilated cardiomyopathy (DCM). In this case, adipose-derived MSCs (ASCs), genetically modified to express stromal-derived factor-1, were administered retrogradely through coronary venous delivery. While the procedure was completed successfully in most cases, one dog died from ventricular fibrillation post-delivery. Despite this complication, the remaining dogs survived without issues, although long-term follow-up indicated continued disease progression with no significant survival advantage over existing treatments for DCM [125].

In contrast to these mixed results, a study investigating the use of human adipose-derived stem cells (h-ASCs) in dogs with myocardial lesions created by radiofrequency ablation offered a more encouraging outcome. The h-ASCs successfully homed to the damaged myocardial tissue and expressed cardiomyocyte markers, suggesting differentiation into heart cells. However, some of the stem cells also accumulated in the lungs and spleen, indicating that further refinement of delivery methods is necessary to optimize cardiac targeting [100]. In a related study, human mesenchymal stem cells (hMSCs) were evaluated for biological pacemaking in dogs with complete heart block. The hMSCs, modified with the mHCN2 gene and injected into the left ventricular myocardium, provided stable pacemaker function over six weeks. No cellular or humoral rejection was observed, demonstrating that hMSCs could be a viable alternative to electronic pacemakers, with the added benefit of catecholamine responsiveness [98].

Similarly, another study investigated the use of hMSCs enhanced to differentiate into cardiac cells for heart tissue repair. Spheroids of these modified hMSCs were seeded onto extracellular matrix patches and implanted into dogs with right ventricular defects. The spheroid-derived cells expressed cardiac-specific proteins, exhibited functional calcium currents, and demonstrated sarcomere-like striations, leading to improved regional heart function compared to dogs receiving unmodified hMSCs [99]. In a complementary approach, another study focused on cardiosphere-derived cells (CDCs), which are progenitor cell clusters derived from cardiac tissue. When infused into Doberman pinschers with spontaneous dilated cardiomyopathy (DCM), these cells were shown to reduce scar size, improve myocardial viability, and enhance cardiac function without adverse effects or signs of immune rejection [97]. Together, these studies underscore the promise of both cardiac-specific differentiation and tissue-originated stem cell approaches in promoting cardiac regeneration and repair in canine models.

Building on this, a canine infarct model demonstrated the benefits of combining basic fibroblast growth factor (bFGF) with MSCs delivered via retrograde coronary venous infusion. This combination significantly improved cardiac repair compared to MSCs or bFGF alone, resulting in increased left ventricular ejection fraction, improved vascular density, and reduced apoptosis. These findings suggest that combining MSCs with growth factors like bFGF could be a promising strategy for enhancing cardiac repair following ischemic injury [103]. However, not all studies have yielded such favorable results. For example, mesenchymal stromal cells (MSCs) were evaluated for their potential to enhance bone marrow (BM) graft survival in dogs that received reduced-dose total body irradiation (TBI). Despite strong immunosuppressive properties in vitro, the MSCs failed to prevent graft rejection, highlighting the need for further research into optimizing MSC efficacy in such contexts [125].

In another canine chronic ischemia model, MSCs were tested for their ability to improve cardiac function. After 60 days, the MSC-treated group showed significant improvements in left ventricular ejection fraction compared to controls, along with reduced fibrosis and increased vascular density. However, the MSCs did not differentiate into cardiomyocytes, indicating that while MSCs can enhance vascularization and repair, their role in direct cardiomyocyte replacement remains limited [60]. This aligns with findings from a histological study on myocardial infarction healing in dogs, where MSCs delivered transendocardially or intracoronarily reduced necrosis and increased extracellular matrix deposition but did not differentiate into cardiac cells. These results suggest that MSCs primarily support myocardial healing through paracrine effects rather than direct cell replacement [105].

Lastly, a study on dogs with congestive heart failure secondary to myxomatous mitral valve disease assessed the intravenous administration of allogeneic Wharton’s jelly-derived MSCs. While the treatment was safely administered, no significant improvements in echocardiography, cardiac biomarkers, or survival times were observed compared to the placebo group. The MSC-treated group did, however, show a reduction in lymphocyte and eosinophil counts, indicating some immunomodulatory effects, though the overall functional benefits were minimal [32]. Table 2 summarizes key comparative studies on stem cell therapies specifically for cardiac diseases in dogs, detailing the types of stem cells used, the number of animals treated, dosages, and observed outcomes.

## 5. Future Directions and Recommendations

### 5.1. Research Gaps

Stem cell therapy holds promising potential for treating cardiovascular diseases in small animals, but several gaps in knowledge and research must be addressed to optimize its therapeutic outcomes. Key areas of uncertainty and unanswered questions remain, which provide directions for future research:

#### 5.1.1. Mechanisms of Action and Differentiation

Understanding the mechanisms through which various types of stem cells (SCs) facilitate the regeneration of infarcted heart tissue is crucial for advancing and refining novel SC therapies. The regenerative capacity of SCs is attributed to both direct and indirect (paracrine) mechanisms. Direct mechanisms involve the differentiation of injected SCs into cardiomyocytes or endothelial cells, which then integrate into the myocardial tissue to replace lost cells and improve heart function. Conversely, emerging evidence underscores the significance of paracrine signaling, where SCs release factors that mediate tissue repair and functional recovery without direct cell replacement [96,126]. Research should focus on elucidating these mechanisms to enhance therapeutic strategies and improve outcomes for small animal models in cardiology [127].

#### 5.1.2. Cell Homing and Retention

Cell homing is a crucial aspect of stem cell therapy, involving the migration of stem cells to specific tissue niches. For mesenchymal stem/stromal cells (MSCs), known for their plastic adherence and differentiation into various connective tissues, understanding their homing mechanisms is vital for enhancing therapeutic outcomes [128,129]. MSCs can be administered intravenously or directly at injury sites, and their effective homing is necessary for successful tissue repair. Unlike hematopoietic stem/progenitor cells (HSPCs), MSC homing is less well understood, with ongoing debate about whether MSCs localize to tissues due to passive entrapment or active guidance mechanisms [129]. MSC homing involves several steps similar to HSPCs, including tethering by selectins, activation by chemokines, and arrest by integrins, with CD44 playing a significant role in initiating rolling. However, the specific selectins used by MSCs are not well understood, and molecules like galectin-1 or CD24 might serve as ligands for P-selectin on MSCs [130,131,132]. Matrix metalloproteinases (MMPs), including MMP-1, are involved in MSC transmigration across the endothelial basement membrane, while chemotactic signals such as CXCL12, PDGFα, and other growth factors guide MSC migration [133,134]. The expression of various chemokine receptors, such as CXCR4 and CXCR7, highlights the complexity of MSC migration [135,136]. Despite these insights, MSC homing remains inefficient in many cases, and further research is needed to improve our understanding and optimize MSC-based therapies [137,138,139].

While MSC homing plays a crucial role in cardiac regeneration, their limited retention at the target site remains a significant challenge. Scaffold-based delivery systems offer a promising approach to enhance MSC retention and therapeutic efficacy. Recent advancements in biomaterial engineering, such as electrospun polymers, have enabled the development of scaffolds that mimic the extracellular matrix (ECM), providing structural support for MSC attachment and controlled release. Studies have demonstrated that polycaprolactone (PCL)-based scaffolds can facilitate MSC retention and improve vascular remodeling, as evidenced by enhanced luminal expansion and reduced inflammatory responses in vascular applications [140]. Similar strategies could be adapted for cardiac tissue repair, where MSC-laden hydrogels, patches, or wraps may provide localized cell delivery, improve engraftment, and promote myocardial regeneration. Integrating scaffold-based approaches with MSC therapy holds potential for overcoming the current limitations of cell-based cardiac repair by enhancing both homing and retention.

#### 5.1.3. Cell Differentiation and Maturation

Initially, it was believed that transplanted BM-MSCs could significantly aid myocardial recovery by differentiating into cardiomyocytes and integrating with the host myocardium, along with promoting neovascularization [141,142]. However, the ability of adult stem and progenitor cells to differentiate into functional cardiomyocytes remains debated. Some studies have reported that bone marrow-derived mononuclear cells (BM-MNCs) can transdifferentiate into cardiomyocytes, as well as endothelial and smooth muscle cells, contributing to cardiac repair [143,144,145]. Conversely, other research suggests that cell fusion with existing cardiomyocytes is more common [146,147,148]. This controversy extends to peripheral blood (PB) and adipose tissue-derived stem cells, with both transdifferentiation and cell fusion being observed in vivo [149,150]. Data on cardiac stem and progenitor cells are similarly mixed, with some studies indicating direct differentiation into new cardiomyocytes [151,152], while others show equal occurrences of differentiation and cell fusion [153]. The effectiveness and maturity of newly formed cardiomyocytes vary, with some studies demonstrating contractility and maturation within weeks [86,151], while others report a lack of mature cardiomyocytes from transplanted c-kit+ cells even after years [154].

#### 5.1.4. Immunological Considerations

Allogeneic stem cell therapies carry the risk of immune rejection, a critical yet understudied issue in veterinary applications. The immune system’s response to transplanted cells is mediated by both the innate and adaptive arms. The innate immune system, acting rapidly and non-specifically, plays a crucial role in the early rejection of transplanted cells. Natural killer (NK) cells, as part of the innate response, recognize and destroy donor cells lacking self-human leukocyte antigens (HLAs), triggering further immune reactions through cytokine signaling. This process can be exacerbated by culture conditions during cell therapy preparation, leading to the expression of immunogenic molecules [155,156,157]. Conversely, some cells may express inhibitory molecules or cytokines, which could mitigate NK cell-mediated rejection by dampening the immune response [158,159,160].

The adaptive immune system, which is longer-lasting and more specific, also plays a significant role in rejecting allogeneic cells. T cells, upon recognizing donor antigens via major histocompatibility complex (MHC) molecules, activate cytotoxic responses that can lead to graft rejection. This response can occur through direct recognition of donor antigens by recipient T cells or indirectly via recipient antigen-presenting cells (APCs) processing donor antigens [161,162,163]. These pathways contribute to acute and chronic rejection of transplanted stem cells. However, such immune responses might be mitigated by pre-screening donor MSCs for the absence of MHC II expression, reducing the likelihood of T cell-mediated rejection [164].

Recent advances in genetic engineering, including the knockout of HLA genes, have shown promise in reducing immunogenicity by creating universal donor cells that are less likely to trigger both innate and adaptive immune responses [165,166]. However, challenges remain, particularly the activation of NK cells, which require further investigation to develop balanced strategies that target both immune systems [167]. To improve the safety and efficacy of allogeneic stem cell therapies, strategies like genetic modifications to incorporate immune-inhibitory ligands or using immunosuppressive agents are being explored. These approaches aim to induce immune tolerance and protect transplanted cells from immune-mediated destruction [167,168].

Mesenchymal stromal cells (MSCs) exhibit unique immunoregulatory and regenerative properties, positioning them as promising candidates for cellular therapies targeting autoimmune diseases and inflammation. The growing body of clinical and preclinical research is elucidating the molecular mechanisms through which MSCs mediate their therapeutic effects. Notably, MSCs regulate both innate and adaptive immune responses, offering insight into their potential for treating immune-related conditions. Recent studies have demonstrated that MSCs can suppress T cell proliferation, inhibit natural killer T (NKT) and γδ T cells [169], and restore the balance between T helper (TH) 1 and TH2 cells, which may be skewed in inflammatory disorders [170,171]. Furthermore, MSCs promote the induction and survival of regulatory T (TReg) cells, a critical component in maintaining immune tolerance and modulating immune responses [172,173]. The immune modulation by MSCs extends beyond T cell regulation to include the inhibition of dendritic cell (DC) maturation and the suppression of B cell responses. Through secretion of immunomodulatory cytokines such as interleukin-10 (IL-10) and transforming growth factor-beta (TGF-β), as well as bioactive metabolites like nitric oxide and prostaglandin E2, MSCs orchestrate a complex immune environment that supports tissue repair and reduces inflammation. These properties suggest that MSC-based therapies may offer effective treatments for autoimmune diseases, where immune dysregulation plays a pivotal role [173].

Moreover, the concept of immune privilege associated with allogeneic MSCs is being revisited. Initially thought to avoid immune rejection, MSCs express major histocompatibility complex (MHC) class I molecules and can upregulate class II under inflammatory conditions, indicating a more nuanced interaction with the immune system. While MSCs may still hold promise for certain clinical applications, their immune compatibility, especially in allogeneic settings, remains an area requiring further investigation to optimize therapeutic outcomes [43].

MSCs’ ability to modulate both innate and adaptive immune responses presents a compelling rationale for their use in treating autoimmune and inflammatory diseases. However, understanding the intricate balance of immune system interactions and the factors that influence MSC engraftment and longevity will be crucial for enhancing the efficacy of MSC-based therapies [174].

#### 5.1.5. Comparative Effectiveness of Stem Cell Sources

MSCs can be derived from a wide range of sources, each offering distinct advantages and challenges. BM-MSCs were traditionally the most commonly used source for clinical applications, but ADSCs and UC-MSCs have gained attention due to their broader availability and ease of collection [175,176,177]. Despite the invasive nature of isolating BM-MSCs and ADSCs, these autologous sources are still commonly used. However, UC-MSCs, which are less invasive to obtain and can be cryopreserved for future use, present a compelling alternative due to their higher proliferation capacity and differentiation potential [177,178,179]. This tissue source-associated heterogeneity suggests that each MSC type may offer different therapeutic benefits depending on the specific clinical context.

The comparative effectiveness of MSC sources largely depends on factors such as differentiation potential, paracrine effects, and the ability to promote angiogenesis. For instance, UC-MSCs are particularly rich in angiogenic factors, making them a promising option for regenerative therapies focused on vascular repair [180]. In contrast, ADSCs are easier to harvest and can be collected in larger quantities compared to BM-MSCs, offering practical advantages in regenerative medicine. Future studies should further explore the tissue-specific characteristics of these MSCs, especially with regard to their therapeutic potential in cardiac conditions [181,182]. Additionally, induced pluripotent stem cells (iPSCs) have the ability to generate large numbers of MSC-like cells in vitro, which could help overcome some of the limitations associated with adult tissue-derived MSCs [183,184].

As research continues to investigate the differences between MSC sources, a more precise understanding of their clinical applications will emerge. The need for standardized protocols, including methods of isolation, cryopreservation, and potency assays, remains critical for ensuring the safety and effectiveness of MSC-based therapies [185,186,187]. The future of MSC research lies not only in identifying the most suitable stem cell source but also in refining the processes related to stem cell isolation, expansion, characterization, and delivery methods, which are crucial for their effective and safe clinical use [188].

### 5.2. Technological Advances

Recent technological advances are significantly transforming stem cell therapies in small animal cardiology, offering novel approaches to address the limitations of traditional treatments. One such advancement is tissue engineering, which leverages the creation of 3D scaffolds that mimic the native extracellular matrix (ECM). These scaffolds act as temporary structures that promote cell proliferation and vascularization, facilitating the formation of new cardiac tissue [189,190]. By providing an environment conducive to cell growth, scaffold technology can support the regeneration of damaged heart tissues, offering a promising route to improve cardiac function post-injury [191,192,193].

The extracellular matrix (ECM) plays a crucial role in enhancing MSC proliferation, adhesion, and vascularization by providing biochemical and structural support. Natural polymers within the ECM, such as polysaccharides and proteins, contribute to MSC retention and therapeutic efficacy by activating key signaling pathways, including FAK/Src, MAPK, PI3K/Akt, Wnt/β-catenin, and YAP/TAZ [194]. These pathways regulate cell survival, differentiation, and paracrine secretion, ultimately improving MSC-mediated tissue regeneration. Recent studies suggest that integrating electrospun scaffolds with biological polymers can optimize MSC delivery and functionality, combining the mechanical stability of synthetic materials with the bioactivity of natural ECM components [194].

Gene editing and reprogramming technologies have also emerged as powerful tools in regenerative medicine. Techniques like CRISPR/Cas9 enable the correction of genetic defects that may cause cardiac dysfunction. Additionally, reprogramming somatic cells into induced pluripotent stem cells (iPSCs) holds significant potential, allowing for the production of patient-specific stem cells that avoid immune rejection issues often associated with allogeneic MSCs transplants [195,196,197]. This approach can revolutionize the treatment of various cardiovascular diseases by enabling the generation of personalized stem cells capable of differentiating into cardiomyocytes [198].

Nanotechnology further enhances stem cell therapy by facilitating precise delivery and control of therapeutic agents. Nanoparticles can be used to label stem cells, track their migration, and improve their retention at injury sites. By influencing the cellular microenvironment, nanomaterials help regulate stem cell behavior, promoting effective tissue repair and regeneration [199,200,201]. This technology is valuable for cardiac regeneration, where ensuring the proper integration of stem cells into damaged heart tissue is critical [202].

Another area of advancement involves the use of biomaterials and bioengineered tissues. Biocompatible materials such as bio-glass grafts and engineered tissues resembling native organs offer innovative solutions for cardiac repair. These materials provide structural support while integrating into the healing process, enhancing the body’s natural regenerative capabilities [203,204]. The combination of biomaterials with stem cell technology is poised to significantly improve the outcomes of stem cell therapies in small animal cardiology.

Emerging technologies such as heart organoids offer a promising alternative for heart transplantation [205,206,207,208]. These mini-organs, generated from stem cells, mimic the structure and functionality of the heart, providing a powerful tool for disease modeling, drug testing, and potentially for regenerative medicine. Heart organoids hold great potential in studying complex cardiac conditions, such as myocardial infarction, and in developing personalized therapeutic interventions. Their ability to replicate native heart tissue in vitro offers hope for advancing stem cell therapies, as they provide an intricate platform for understanding cardiac development and pathophysiology [209,210,211]. Emerging trends, challenges, and future directions in stem cell therapies are detailed in Table 3, providing insights into cell sources, delivery methods, mechanisms of action, and technological advances.

## 6. Conclusions

Stem cell therapies hold transformative potential for canine cardiology, offering innovative approaches to cardiac repair through immunomodulation, angiogenesis, and tissue regeneration. This review highlights the comparative efficacy of MSCs from bone marrow, adipose tissue, and Wharton’s jelly, alongside cardiac stem cells, emphasizing their unique therapeutic benefits and challenges. Emerging trends, such as genetic modifications, combination therapies, and advanced delivery methods, enhance cell retention, survival, and functional integration. However, variability in therapeutic outcomes underscores the need for standardized protocols and robust preclinical models. Clinical integration requires addressing regulatory, technical, and ethical challenges through interdisciplinary collaboration and large-scale trials. By aligning scientific innovation with practical veterinary needs, stem cell therapies are poised to revolutionize the management of canine cardiovascular diseases, shifting from palliative care to true myocardial regeneration.

## Figures and Tables

**Table 1:** Characteristics and comparative efficacy of different stem cell types.

Type of Stem Cells	Accessibility	Clinical and Preclinical Efficacy	Safety	Mechanism of Action
Bone Marrow-Derived Stem Cells (BM-MSCs)	Harvested from bone marrow, invasive procedure.	Demonstrated improvement in cardiac function, reduced infarct size, and increased vascularization in ischemic heart disease [60,94].	No major safety concerns were reported.	Differentiation into cardiomyocytes, smooth muscle, endothelial cells; paracrine signaling [44,96].
Adipose-Derived Stem Cells (ASCs)	Easily harvested from subcutaneous adipose tissue.	Improved cardiac function, reduced fibrosis, and enhanced angiogenesis in preclinical models [70,71,72,74].	No evidence of tumorigenicity or arrhythmogenic risk in preclinical/clinical testing [73,75].	Differentiation into endothelial and smooth muscle cells, paracrine effects promote vascularization and tissue survival [73,75].
Cardiosphere-Derived Cells (CDCs)	Requires access to adult cardiac tissue; autologous use possible.	Improved cardiac function in dilated cardiomyopathy (DCM); mixed results in long-term survival [97].	Generally safe, no signs of immune rejection, though more studies are needed [97].	Stimulate endogenous repair through paracrine signaling and promote neovascularization [97].
Wharton Jelly-Derived MSCs (WJ-MSCs)	Easily harvested from umbilical cord tissue, non-invasive.	Effective in immunomodulation but limited efficacy in improving heart function in CHF models [32].	Safe administration with no significant side effects, though therapeutic benefits are modest [32].	Paracrine signaling, angiogenesis, homing to damaged tissues; low differentiation into cardiomyocytes [79,80,81].
Human MSCs (hMSCs)	Generally harvested from bone marrow or adipose tissue.	Effective in biological pacemaking and improving cardiac function; beneficial in chronic ischemia models [98,99].	No cellular or humoral rejection was observed, and catecholamine responsiveness was confirmed in pacemaking studies [98].	Differentiation into cardiac-like cells, and paracrine effects; also effective in gene-modified therapies [98,100].
Deciduous Teeth Stem Cells (pDSCs)	Harvested from puppy deciduous teeth, limited availability.	Significant improvement in cardiac function and quality of life in dogs with chronic valvular heart disease [101].	No significant adverse effects were observed in clinical studies [101].	Paracrine signaling promotes cardiac function; and minimal direct cardiac differentiation [101].
Cardiac Stem Cells (CSCs)	Obtained from cardiac tissue; requires a cardiac biopsy, making it a more invasive procedure.	Shows potential in enhancing myocardial regeneration and improving cardiac function through differentiation into cardiomyocytes and promoting angiogenesis; results vary across studies [87,91].	Generally safe with low immunogenicity, though long-term effects and potential for arrhythmias require further investigation [87].	Differentiates into cardiomyocytes and vascular cells; releases paracrine factors that stimulate resident cardiac cells, angiogenesis, and tissue repair [87,90,91].

**Table 2:** Comparative studies on stem cell therapies for cardiac diseases in canine cardiology.

Condition	Type of Cells	Number of Animals	Dose of Cells	Route of Administration	Frequency of Treatments	Evaluations	Results
Ischemic heart disease [60]	Bone marrow-derived stem cells (BM-MSCs)	12	100 × 10^6^ MSCs/10 mL saline	Intramyocardial injections	Single dose	Resting and stress 2D echocardiography	Increased vascularity, and improved cardiac function through smooth muscle and endothelial cell differentiation.
Chronic myocardial infarction [94]	Bone marrow mononuclear cells (BMNCs)	24	227 ± 32 × 10^6^ MSCs and 232 ± 40 × 10^6^ BMNCs	Intramyocardial injections	Single dose	Echocardiographic analysis	Improved cardiac contractility, regional systolic function, reduced infarct size, and increased angiogenesis.
Chronic Chagas cardiomyopathy [109]	Autologous BM-MSCs	5	100 × 10^6^ MSCs	Intracoronary injection	Single dose	Electrocardiography, echocardiography	Significant improvement in peak velocity of aortic flow.
Chronic valvular heart disease [101]	Deciduous teeth stem cells (pDSCs)	20 client-owned	1 × 10^6^ cells of pDSC/kg B.W.	Intravenous injections	Two injections on day 0 and day 14 following the initial pDSCs administration.	ECG, echocardiography, radiography, blood pressure	Improved LVEF and quality of life scores; the control group showed no significant improvement.
Non-ischemic dilated cardiomyopathy (DCM) [97]	Cardiosphere-derived cells (CDCs)	8 with spontaneous DCM	30 million allogeneic CDCs	Via coronary vessels	Single dose	Echocardiography, histology (12-month follow-up)	No adverse events, no immune rejection, no significant survival advantage.
Dilated cardiomyopathy [107]	Adipose-derived MSCs (ASCs) transduced with stromal-derived factor-1	15	1 × 10^7^ cells suspended in 20 mL PBS	Retrograde coronary sinus infusion	Single dose	ECG, echocardiography, Holter monitoring (2-year follow-up)	The procedure was safe; no survival advantage over existing treatments; one dog developed ventricular fibrillation and died.
CHF secondary to MMVD [32]	Wharton jelly-derived MSCs (WJ-MSCs)	10	2 × 10^6^ cells/kg IV	Intravenous injections	Three injections administered three weeks apart.	Echocardiography, ECG, cardiac biomarkers	Lymphocyte and eosinophil counts decreased, with no significant improvement in echocardiography or survival.
Myocardial infarction [105]	Allogeneic MSCs	7	100 × 10^6^ DAPI-labeled MSCs	Transendocardial (TE) or intracoronary (IC).	Single dose	Histopathology after 21 days	MSCs reduced necrosis, increased extracellular matrix deposition; no cardiac differentiation.
AV block [123]	mHCN4-modified canine MSCs	-	3.0 to 3.5 × 10^6^ viable cells	Subepicardial injection into left ventricular anterior wall	Single dose	Heart rate variability (HRV), cardiac parameters (6 weeks), histology, Western blot	Improved maximum heart rate and impulse generation at the injection site, stable heart rate by Week 4, increased HRV during exercise, survival of modified cMSCs, HCN4 expression confirmed in heart tissues.
Coronary ischemia [124]	Endothelial progenitor cells (EPCs), BM-MSCs	9	35 × 10^6^ cells/kg heart weight	Intracoronary infusion	Single dose	Electrocardiogram, cardiac enzyme tests, echocardiography, histopathology	Safe and feasible procedure with no significant changes in vital signs, ECG, cardiac function, or heart histology
Radiofrequency ablation [100]	Human adipose-derived stem cells (h-ASCs)	14	1 × 10^7^ cells	Intravenous injections	Single dose	Prussian blue staining, immunohistochemistry, flow cytometry	h-ASCs homed to injured atrial tissue, expressed cardiomyocyte-like markers (α-actinin, troponin-I, connexin 43, VEGFR-2); no immunorejection or neoplasm-like cells; h-ASCs also detected in lungs and spleen.
Complete heart block [98]	Human MSCs (hMSCs)	-	≥700,000 hMSCs	subepicardial at 3 closely apposed sites in the left ventricular anterior wall	Single dose	Pacemaker function, catecholamine responsiveness, histology, rejection studies	Stable pacemaker function for 6 weeks, catecholamine-responsive rhythms, no cellular or humoral rejection, doses >700,000 hMSCs sufficient for stable impulse generation.
Right ventricular defect [99]	Spheroid-derived human mesenchymal stem cells (hMSCs)	-	250,000 hMSCs were used to form each spheroid. 2 million hMSCs were used for comparison in the experiment	The cells were placed on a scaffold (ECM) and implanted into the heart tissue	Cells were cultured for 3 days to form spheroids and then for 7–10 days before implantation	Calcium channel analysis, cardiac protein expression, mechanical function evaluation, histology	hMSC spheroids expressed cardiac-specific proteins (α-actinin, cardiotin, ANP), showed functional calcium currents, and improved regional mechanical function compared to unmanipulated hMSCs.
Chronic myocardial infarction [90]	Autologous c-kit–positive cardiac stem cells (CSCs)	19	1 × 10^6^ cells/mL concentration. 0.8 mL of the cell suspension	Intramyocardial injection	Single dose	Cardiac MRI at 6 and 30 weeks post-infarct, LV volumes, ejection fraction	Attenuation of adverse left ventricular remodeling, reduced increase in end-systolic volume, and preservation of left ventricular ejection fraction over 30 weeks.

**Table 3:** Emerging trends, challenges, and future directions in stem cell therapies.

Category	Current Trends	Challenges	Future Directions
Cell Sources	Increasing use of MSCs from bone marrow, adipose tissue, and Wharton’s jelly [175,176,177].	Limited differentiation into cardiac cells; ethical concerns with some cell types [176].	Explore genetically modified stem cells, induced pluripotent stem cells (iPSCs), and engineered cells for enhanced regeneration [166,186].
Delivery Methods	Retrograde coronary venous delivery, intracoronary injections, and intramyocardial injections are common [112,113]	Risks of cell embolization, inefficient homing, and invasiveness of procedures [110,124].	Development of less invasive delivery systems such as targeted nanoparticles or scaffold-based cell delivery [202].
Mechanism of Action	Emphasis on paracrine signaling for angiogenesis, anti-fibrosis, and immune modulation [96,142].	Limited direct differentiation into functional cardiomyocytes; poor long-term engraftment [154].	Enhance cell survival and efficacy by preconditioning, and genetic engineering to increase paracrine factor secretion (e.g., exosomes, growth factors) [197,199].
Preclinical/Clinical Efficacy	Positive results in improving cardiac function in myocardial infarction and heart failure models [128].	Heterogeneous responses across species, lack of long-term data, and modest improvements in survival [125].	Conduct large-scale, standardized trials across species and explore combination therapies (stem cells + drugs/growth factors) [121].
Stem Cell Tracking and Retention	Imaging techniques like MRI and SPECT are used to track cells post-delivery [137,183].	Poor retention of stem cells at the target site; rapid clearance or apoptosis post-injection [139].	Investigate scaffolds, hydrogels, and biomaterials to enhance cell retention and integration with host tissue [189,191].
Combination Therapies	Combining MSCs with growth factors, gene therapy, or cardiac stem cells [212,213,214,215].	Lack of optimized combinations, and unclear mechanisms of synergistic effects [117].	Further explore synergies between stem cells and adjunctive therapies (e.g., CRISPR gene editing, biomaterials) [126,198].
Ethical and Regulatory Concerns	Autologous stem cell therapies reduce ethical concerns, and allogeneic therapies gaining traction [119].	Regulatory hurdles for new therapies, long and expensive approval processes, and lack of standardization [120].	Develop global regulatory frameworks and ethical guidelines for stem cell therapies in veterinary medicine [122].
Technology Integration	Advances in tissue engineering (3D scaffolds, bio-printed tissues) and nanotechnology [203,204].	The complexity of integrating technologies into routine clinical practice; high costs [204].	Leverage bioengineering advances to create “off-the-shelf” stem cell products for cardiac repair [209].
Patient Selection and Stratification	Identifying specific subgroups (e.g., age, disease stage) that benefit most from stem cell therapies [216].	Variability in disease progression, and patient-specific responses to treatment [99].	Personalize stem cell therapies based on genetic, molecular, and disease profiles for optimized outcomes [180].

## Data Availability

The collected literature is available on request.
